# The Acceptability and Feasibility of Using Text Messaging to Support the Delivery of Physical Health Care in those Suffering from a Psychotic Disorder: a Review of the Literature

**DOI:** 10.1007/s11126-020-09847-x

**Published:** 2020-09-24

**Authors:** Henry Griffiths

**Affiliations:** Cardiff University School of Medicine, Cardiff, UK

**Keywords:** Text messaging, Telemedicine, Physical health, Psychotic disorder

## Abstract

Those suffering with serious mental illness (SMI), such as psychotic disorders, experience life expectancy 15 years shorter than the general population. Cardiovascular disease is the biggest cause of death in those with psychotic disease and many risk factors may be limited by healthy lifestyle choices. Text messaging interventions represent mobile health (mHealth), a nascent way to deliver physical health care to those suffering with a psychotic disorder. This paper aims to review the literature on the feasibility of text messaging to support the delivery of physical health care in those with a psychotic disorder. A thorough electronic database literature review of Medline via Ovid, Embase, APA Psycinfo, Scopus, Cochrane and Web of Science was conducted. Articles were included if text messaging was used as an intervention targeting the physical health of patients with psychotic disorders. A final sample of 11 articles satisfied the eligibility criteria, of which, 3 were ongoing randomised controlled trials. Of the 8 completed trials, all demonstrated the promising feasibility of text messaging, assessed via quotes, conversation samples, response rates, questionnaires or directly based on physical results. 36% of studies analysed those with schizophrenia or schizoaffective disorder, 55% with SMI and 9% with schizophrenia and psychotic disorders, mood disorders or anxiety disorders. Text messaging was used as motivation or reminders (91%), service delivery (27%) or social support (27%) with studies targeting multiple themes simultaneously. This review highlights compelling evidence for the feasibility of text messaging for improvement of physical health in those suffering with psychotic disorders.

## Introduction

The health disparity experienced by those suffering with psychotic disorders is well defined and unacceptable. Life expectancy has been shown to be approximately 15 years shorter, with an average mortality rate 2 to 3 times higher, than the general population [1]. The reason for this is likely multifactorial and studies have demonstrated links between physical inactivity [2, 3], tobacco consumption [4], dietary insufficiency and the adverse metabolic effects of antipsychotic medication [3] in those with serious mental illness (SMI). These contribute collectively towards obesity rates that are almost double that of the general population [5] and significantly raised cardiovascular disease (CVD) risk [6], which is particularly worrying given that CVD is the single biggest cause of death in those with psychotic illnesses [7].

SMI includes those with psychotic disorders such as schizophrenia spectrum disorders (SSD) and bipolar disorder (BD), as well as major depressive disorder (MDD) [8]. In the United Kingdom (UK), clear guidelines have been set that recommend that people with a psychiatric condition have access to both medical care and interventions to promote a healthy lifestyle [9]. Despite promising health outcomes and improved quality of life with lifestyle interventions in those with SMI [10–12], these are difficult to replicate in practice due to the substantial workforce, organisational and financial resources required [13, 14]. Technological implementation, such as the nascent mobile health (mHealth), could offer a method of improving healthcare delivery without the significant cost. Smartphone ownership has been reported as high as 72–93% in those with SMI [15, 16] and is suspected to rise further [17], with mobile phone ownership almost ubiquitous at 97% [18]. Text messaging has not only been found to be efficacious [19, 20] and cost-effective [21], but is also unique in that it permits interventions in real time and in almost any location [22].

Despite emerging research being published looking at the use of text messaging within mental health, research is lacking targeting the physical health problems within patients with SMI specifically. In this literature review, the current and future prospects of mHealth within psychotic disorders is challenged through inspecting the literature for the feasibility of using text messaging as an intervention to support the delivery of physical health care.

## Methods

### Search Strategy

An extensive search of Medline via Ovid, Psycinfo, Embase, Scopus, Cochrane and Web of Science peer-reviewed electronic databases was conducted from April to May 2020. A list of keywords was created around the three subject headings ‘text messaging’, ‘physical fitness’ and ‘schizophrenia spectrum and other psychotic disorders’ (Appendix).

### Eligibility Criteria

The inclusion criteria were as follows, with exclusion criteria as the reverse of each point:

- Original research articles: reviews, letters and frontline reports were excluded
- Completed or ongoing research
- Use of text messaging as an intervention
- Measure of physical activity or health
- Study populations included those with schizophrenia spectrum and other psychotic disorders
- Study outcome included the feasibility, acceptability, usability, efficacy or effectiveness
- Available in English

### Data Extraction and Analysis

The database search, screening and later analysis were performed independently by the sole author (HG). Initially, titles and abstracts were screened before relevant article full-text reviewing was performed. Only publications that satisfied the eligibility criteria in full made up the final literature review sample.

The following information was extracted from the final sample: study design, location, sample size, duration including follow up, population characteristics including mental health condition(s) assessed, the methodology including intervention and control (if applicable), the technological features, the outcomes and their method of measurement and the main findings.

## Results

**Fig. 1:**
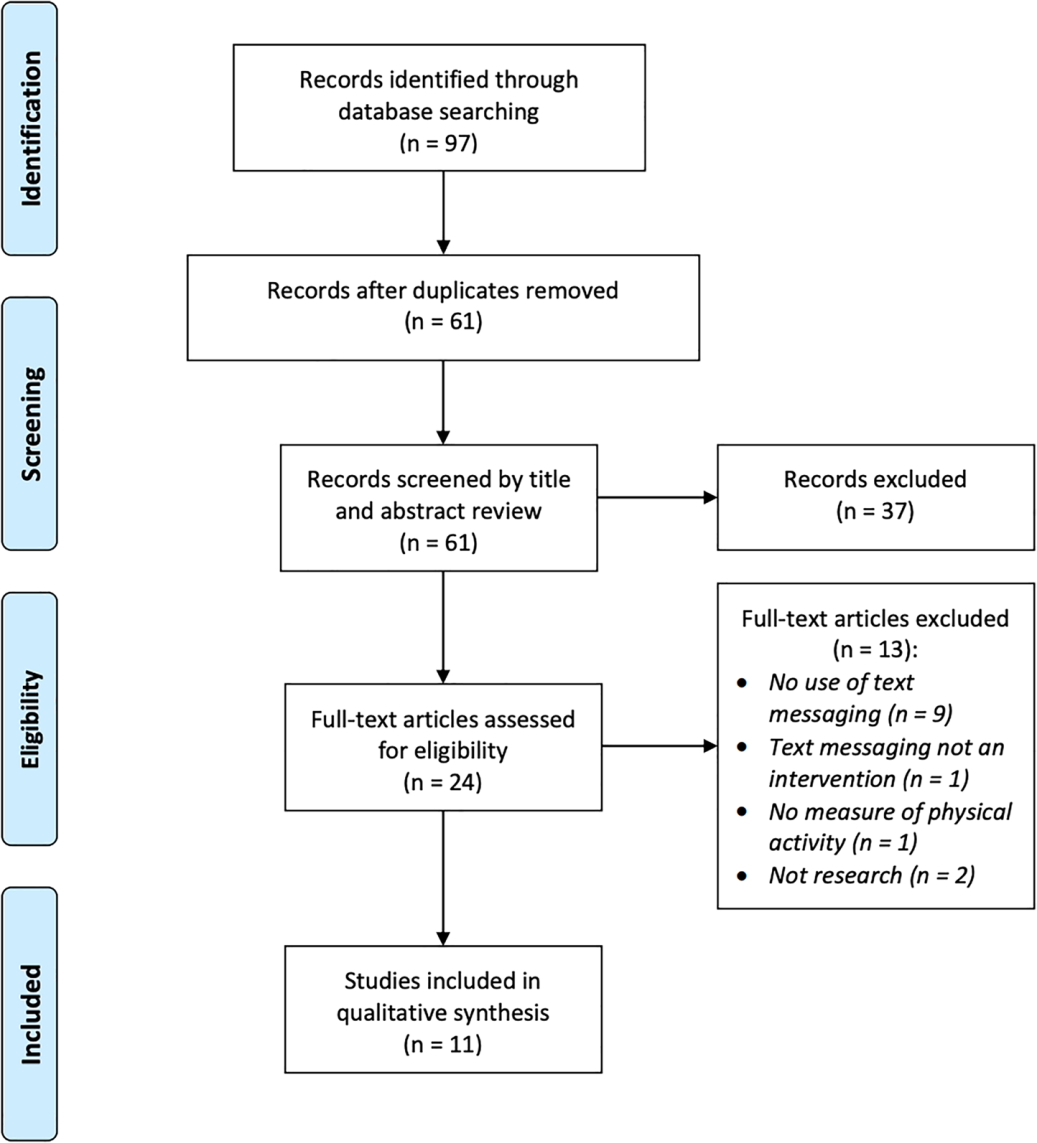
PRISMA flow diagram of the literature review process [23]

The literature search initially returned 97 results (Fig. 1). After removal of duplicates, 61 records were screened by title and abstract review. 24 article full-texts were then reviewed, with exclusions due to the articles not using text messaging at all or not as the chosen mHealth intervention (*n* = 10), not measuring physical activity or health (n = 1) or not being original research papers (*n* = 2). A sample of 11 records meeting the eligibility criteria was finalised.

### Study Characteristics

Concerning the study types, 18% were qualitative (*n* = 2) [24, 25], 27% were cross-sectional (*n* = 3) [26–28] and 36% were randomised controlled trials (RCTs; *n* = 4) [29–32], with 18% classifying for both qualitative and cross-sectional (n = 2) [33, 34]. There was large variability in study duration, which ranged from 4 weeks [33] to 12 months [29, 30, 32]. Other study lengths included 12 weeks [24, 25, 31], 16 weeks [28], 24 weeks [34], 3 months [27] and 6 months [26]. Due to the nature of most articles assessing early feasibility of mHealth, the majority of studies had sample sizes below 34: *n* = 8 [24], *n* = 10 [33], *n* = 11 [28, 34], *n* = 15 [31], *n* = 17 [25], *n* = 34 [26], *n* = 144 [30], *n* = 204 [29], *n* = 267 [27], *n* = 526 [32]. 64% of studies drew from populations based in the United States of America (USA; *n* = 7) [24–27, 30, 33, 34], 18% in Taiwan (n = 2) [28, 31], 9% in Spain (n = 1) [29] and 9% in Pakistan (n = 1) [32].

With regard to the mental illnesses assessed, 36% of studies included patients diagnosed with schizophrenia or schizoaffective disorder (*n* = 4) [25, 28, 31, 32], 55% included patients diagnosed with SMI and thus inclusive of SSD, MDD and BD (*n* = 6) [24, 26, 27, 29, 33, 34] and 9% of studies included patients diagnosed with schizophrenia and psychotic disorders, mood disorders or anxiety disorders (*n* = 1) [30]. One study specifically required participants to have a dual diagnosis of schizophrenia or schizoaffective disorder and substance abuse [25].

All studies solely included those aged 18 or above, besides one ongoing RCT, which extended the lower age range to 17 [32]. The oldest age range accepted was 40–70 [29] and the widest 21–77 [27]. One study looked specifically at young adults aged 18–35 [30] and another specifically at older adults with chronic health conditions resulting in a mean age of 68.8 [24]. 27% of studies looked specifically at mentally ill populations with comorbid obesity classified as body mass index (BMI) ≥25 [30] or BMI ≥30 [26, 34]. One ongoing study is targeting a population of smokers with mental illness, classified as active smokers of ≥10 cigarettes per day and cumulatively ≥10 packets per year [29]. In studies with specific gender demographics mentioned (*n* = 6) [24, 27, 28, 31, 33, 34], 66% of study participants were women. In those that released ethnic demographics (*n* = 4) [24, 27, 33, 34], 93% were white.

### Use of Technology

As per the inclusion criteria, text messaging was incorporated into the study design of all studies. One-way text messaging refers to the sole receiving of messages by participants, whereas two-way text messaging allows participant replies. One study directly compared one-way versus two-way text messaging [31]. Other studies focused on one-way [26, 28–32, 34] or two-way [24, 25, 31, 33] alone. Two-way text messaging was further subdivided into conversing between patient and staff [25, 31, 33] or peer-to-peer [24].

The type of text message intervention was characterised into three themes: motivational/reminder, service delivery and social. 91% of the articles incorporated motivational/reminder text messages into the intervention methodology (*n* = 10) [24–26, 28–34]. 27% also achieved healthcare service-delivery through text messaging (*n* = 3) [25, 31, 33] and 27% further focused on the social aspect of text messaging (n = 3) [24, 31, 33].

Non-text messaging technology found within the studies included Facebook groups for peer-to-peer encouragement [30, 34], smartphone applications (apps) for progress tracking, messaging services and self-reporting [24, 33], and wearable technology to track physical activity and encourage self-reporting [26, 30, 34].

### Outcomes Assessed

The feasibility, acceptability, effectiveness or usability of text messaging as an intervention for improving the physical health of those with SMI, or willingness of participants to use text messaging as an intervention, was assessed in 100% of the studies (*n* = 11) [24–34]. Table 1 shows the evidence provided for feasibility, assessed in all completed trials (*n* = 8). This was measured based on quotes [24], text message conversation samples [25], text message response rate and mobile app usage [33] or questionnaires [27, 28, 31, 33, 34]. Other articles directly measured feasibility based on physical results, including spirometry [29], amount of exercise per week [28], step count [26, 31], medication compliance [32] or cardiovascular risk reduction [30].

**Table 1:** A table to show the outcomes measured and their corresponding evidence for completed trials

Citation:	Outcome(s):	Evidence:
[24]	Feasibility Effectiveness	Patient quotes
[25]	Willingness	Text message exchanges Lifestyle behaviours related to diet, physical activity and sleep were primary topics of conversation
[26]	Feasibility	Higher step counts correlated with greater weight loss: every 1000 step/day increase correlated with a 1.78lbs (0.81 kg) weight decrease (*p* = 0.0314) Wearable worn on 86.2% of study days
[27]	Willingness Feasibility	94.8% smartphone ownership 89.5% mainly used smartphone for text messaging 82.2% willing to text message 100% belief in possibility of use to enhance services Only 58.4% believed that smartphone based interventions used daily could be used to cause positive changes in the consumer’s mental and physical health
[28]	Feasibility Acceptability	Average of 4.6 days/week exercised in home 8 weeks (target 3 days) Average 73 mins/week (target 90 mins) = 81% target No SMS sent: 24.3/40 days exercised = 61% SMS sent: 11.2/16 days exercised = 70% 80% agreed that SMS reminders could support exercise at home 70% agreed that receiving SMS messages didn’t bother them 80% agreed that they regularly read the SMS reminders sent to them
[31]	Feasibility Acceptability Effectiveness	Two-way text message group showed a significant increase in step count/day compared to baseline at week 6 (+1804.8 steps, *p* = 0.043) and week 11 (+2551.7 steps, p = 0.043) 73% said text messaging reminders helped them initiate walking 80% said receiving text messages didn’t bother them 86% said sending text messages didn’t bother them
[33]	Usability Acceptability	Text messaging app used on 94% of study days 98% response rate to messages sent by staff All patients reported moderate to high motivation to exercise at the end of the study Text message conversations were the most favoured aspect of the app
[34]	Feasibility	36% met criteria for statistically significant reduction in CV risk At 6 months, 45% below baseline weight, 45% improved fitness 89% satisfaction with programme, including mHealth and Facebook 89% thought activities were useful 78% thought programme helped them move towards health goal Wellness peer support praised Participants appreciated the text message reminders and found those that encouraged physical activity to be particularly helpful and motivating

Anthropometric measurements, including height, weight, BMI and waist circumference, were taken as supporting data in 64% of studies (n = 7) [26, 28–32, 34]. Cardiovascular fitness was measured in 45% (*n* = 5) [26, 28, 30, 31, 34] and the same studies, with the addition of [29], also measured physical activity directly (55%, *n* = 6). Only one study published significant physical health results, demonstrating that every 1000 steps per day increase correlated with a 1.78lbs weight decrease (*p* = 0.0314) [26]. Although no other significant results were published, one trial recorded a 45% decrease in baseline weight and improvement in cardiorespiratory fitness, measured by the 6 min walk test (6MWT), after a 6 month intervention [34]. However, *p*-values were not stated.

### Ongoing Trials

27% of the studies reviewed were ongoing RCTs (*n* = 3) [29, 30, 32]. The first is measuring weight loss and cardiovascular fitness in order to calculate cardiovascular risk reduction, with text messaging as an aspect of the intervention [30]. Text messaging is not being directly assessed as it is a component of both intervention arms, although it may be inferred as feasible if cardiovascular risk reduction is observed. Another is assessing the feasibility of sending text messages to a patient supervisor to assess medication adherence [32]. The final ongoing RCT is measuring lung damage by spirometry and the effectiveness of anti-smoking text messages by smoking cessation (or reduction), which will be confirmed by carbon monoxide oximetry [29].

## Discussion

This review of 11 peer-reviewed articles appraised the feasibility and acceptability of using text messaging to provide physical health care for those suffering with psychotic disorders. Collectively, the evidence suggests that text messaging may be successfully used as an mHealth intervention within this population. Of the 11 research articles reviewed, 8 were completed, which all produced positive results (Table 1). Most studies provided the patient perspective of mHealth feasibility through a questionnaire.

### Main Findings

Text messaging as a successful intervention may be sectioned with each barrier needing to be overcome in order to suggest feasibility. The first barrier is mobile phone ownership among those with SMI, which has been previously described as high as 98% [18]. The studies in this review did not look specifically at mobile phone ownership as it has been so well documented already. However, smartphone ownership among certified peer specialists in SMI was reported as high as 94.8% [27], although this came from an online survey so respondents may have been more likely to own a smartphone.

The second barrier is the willingness of patients, practitioners and peer specialists to use text messaging as a means of communication. This was demonstrated qualitatively [24, 25] and quantitatively, highlighted by a 98% response rate to messages sent by staff [33], 89% satisfaction scores [34] and 100% belief among peer specialists that text messaging could enhance psychiatric services [27] (Table 1). However, studies in this review looked solely at the perspectives of patients or peer specialists. Concerning two-way text messaging, research is required identifying the willingness of clinicians to partake in this arguably intrusive method of healthcare delivery.

The third barrier is the effectiveness of text messaging at improving the physical health of those with psychotic disease. Aschbrenner et al., reported a decrease from baseline weight in 45% of study participants and a 45% increase in cardiorespiratory fitness, with 36% meeting the criteria for a statistically significant reduction in CVD risk within 6 months [34]. Another study described text messaging facilitating participants exercising at home an average of 4.3 days per week during an 8 week intervention [28]. Text message reminders caused an increase of days exercised on from 61% to 70% [28]. Although non-significant, this is a move in the right direction and larger trials could precipitate more significant results. The type of text messaging intervention must also be considered. Two-way text messaging was shown to be superior to one-way as demonstrated by significant increases in step count per day compared to baseline at week 6 (+1804.8 steps) and 11 (+2551.7 steps) of one study, with no significant results shown in the one-way text group [31].

The fourth barrier is the cost-effectiveness and organisational requirements of the intervention as a whole, including financial expense and clinician time logistics. Mobile technology interventions were described as inexpensive within articles reviewed [28, 29], but this was not examined directly within the trials. In other research, mHealth has been described as a fraction of the cost of physical clinics [35]. Extensive further research is required to overcome this final barrier entirely and classify text messaging as a clinically feasible intervention within psychotic patient management.

### Strengths and Limitations of Reviewed Articles

The studies reviewed clearly demonstrated the feasibility of text messaging in a trial setting. Patients described willingness to engage in text message conversations with clinicians [24, 25, 33, 34] and the potential of communicating with carers to monitor patient medication adherence is currently being assessed [32]. Peer-to-peer support via text messaging emerged as a highly praised intervention technique [24, 27, 28, 31, 34]. This was shown to facilitate development of social ties and allowed patients to feel empowered by their peers, increasing their confidence to make health behaviour changes.

Concerning physical fitness, most studies measured text messaging feasibility rather than physical activity as an outcome, however, certain studies did show promising results [26, 28, 31, 34]. Lifestyle behaviours were evidently of high priority to patients as diet, physical activity and sleep were primary topics of conversation in clinician-patient text exchanges [25]. In clinical practice, thought must be given to the physical activity design plan, as lifestyle physical activity such as walking has been demonstrated as more feasible for patients with SMI than structured exercise [31]. Further, physical activity must be sustainable. Prior studies have demonstrated a significant reduction in health-related outcomes within 2 months of discontinuation of physical activity [36].

The articles reviewed did yield certain limitations. Most notable, was the small sample size used in the majority [24–26, 28, 31, 33, 34] and distinct lack of diversity in study sample populations [26] as predominantly female [24, 27, 31, 34] and of white ethnicity [24, 27, 33, 34]. This severely limits reliability that the study sample is representative of a wider psychotic disordered population. However, one ongoing RCT is looking to combat this by targeting a sample of 50% men and 40% ethnic minority groups within a large sample size (*n* = 144) [30]. One study also looked at a wide range of interventions as one combined group, including text messaging, weight management sessions and exercise classes [34]. This prevented accuracy that the mHealth intervention lead to results due to the presence of confounding variables.

### Future Directions

MHealth is an ever-expanding field and alternative technology may be used synergistically with text messaging to further improve healthcare. Online chat groups demonstrated clear social benefits through peer-to-peer support [30, 34] and an ongoing RCT is investigating the benefits of Facebook groups specifically in the young adult population [30]. This peer social support may have remit for use outside of physical fitness and psychotic disease. Research into their use for those suffering from social isolation and loneliness is encouraged. Wearable technology, usually as a wristwatch, has been well researched with regard to self-reporting and monitoring [26, 30, 33, 34]. However, self-reporting via clinician-patient text messaging for physical activity has not previously been described and should be researched further.

### Strengths and Limitations of the Literature Review

The search, screening and review process of this literature review were conducted by one author and although scrutinous, the opportunity for bias cannot be denied. Many studies reviewed were in the early stages of research, with larger and more diverse studies only in the recruitment phase. This limits reliability of the results in comparison to ongoing trials. Studies were also predominantly located in the USA. Although likely a good representation of western populations, the results are very unlikely to be transferrable to developing countries. The search parameters and eligibility criteria used were precise. This excluded research that may have demonstrated text message feasibility within mental health as a whole and not specifically for physical health. It also limited mHealth interventions to text messaging, so no studies of other technology types were included.

Despite these limitations, this, as far as I am aware, is the first literature review looking specifically at text messaging as an intervention to improve the physical health of those with psychotic disorders. It provides insight into the nascent role of mHealth within psychiatry and should embolden future research in the area.

### Conclusion

The preliminary evidence for the feasibility of text messaging for improving the physical health of those with psychotic disorders is compelling. Text messages permit instantaneous clinician-to-patient or peer-to-peer communication in almost any location. It therefore provides an avenue for improved service delivery and simultaneous social support within personalised medicine. However, due to the small sample sizes and research being in the early stages, we must err on the side of caution with the significance of results. Although promising, it will be more difficult to implement these results clinically. Further research is necessary to evaluate the clinical significance, cost-effectiveness and feasibility in sufficient detail and on a national scale, particularly within the UK.

## Appendix

### Medline via Ovid Search Strategy

1. exp. Text Messaging/
2. exp. Telemedicine/
3. text messag*.mp
4. texting.mp
5. short message service*.mp
6. SMS.mp
7. telemedicine.mp
8. mhealth.mp
9. 1 or 2 or 3 or 4 or 5 or 6 or 7 or 8
10. exp. Physical Fitness/
11. physical health*.mp
12. physical well*.mp
13. physical stat*.mp
14. physical conditio*.mp
15. physical fitness.mp
16. physical health clini*.mp
17. 10 or 11 or 12 or 13 or 14 or 15 or 16
18. exp. Schizophrenia Spectrum and other Psychotic Disorders/
19. exp. Psychotic Disorders/
20. (first episode adj3 psychosis).mp
21. FEP.mp
22. psychosis.mp
23. schizophren*.mp
24. delusio*.mp
25. hallucinat*.mp
26. 17 or 18 or 19 or 20 or 21 or 22 or 23 or 24 or 25
27. 9 and 17 and 26
